# Comorbidity-Associated N-Glycome Profile Shifts: A Preliminary Study Evaluating COPD and Diabetes in Lung Cancer Patients

**DOI:** 10.3390/cancers18213530

**Published:** 2026-11-03

**Authors:** Beatrix Kiss, Rebeka Török, Miklós Szabó, Eszter Csánky, Ilona Slárku, Erika Malyik, Zsófia Robotka, Álmos Kádár, Sara Sardou, Gábor Járvás, András Guttman

**Affiliations:** 1 Research Institute of Biomolecular and Chemical Engineering, University of Pannonia, 8200 Veszprém, Hungary; kiss.beatrix@mk.uni-pannon.hu (B.K.); torokrebeka@gmail.com (R.T.); jarvas.gabor@mk.uni-pannon.hu (G.J.); 2 Department of Pulmonology, Borsod-Abaúj-Zemplén County Central Hospital and University Teaching Hospital, 3526 Miskolc, Hungary; drszabomiklos@bazmkorhaz.hu (M.S.); ecsanky@gmail.com (E.C.); slarku.icu@gmail.com (I.S.); malyikerika@gmail.com (E.M.); zsofia.robotka@gmail.com (Z.R.); kadar.almos@gmail.com (Á.K.); 3 Faculty of Health Sciences, University of Miskolc, 3515 Miskolc, Hungary; 4 Bio-Nanosystems Laboratory, Faculty of Engineering, University of Pannonia, 8200 Veszprém, Hungary; sardou.sarah.blida@gmail.com; 5 Horváth Csaba Memorial Laboratory of Bioseparation Sciences, Center for Translational Medicine, Doctoral School of Molecular Medicine, Faculty of Medicine, University of Debrecen, 4032 Debrecen, Hungary

**Keywords:** lung cancer, comorbidity, chronic obstructive pulmonary disease, diabetes mellitus, serum N-glycome, capillary electrophoresis, CE-LIF

## Abstract

Aberrant glycosylation frequently accompanies malignant, metabolic, and chronic inflammatory diseases; however, it remains unclear how coexisting conditions influence disease-associated alterations in serum N-glycan profiles. To address this question, this preliminary study examined the influence of chronic obstructive pulmonary disease (COPD) and diabetes mellitus (DM) on the N-glycan profiles in 40 patients with lung cancer (LC), divided into four groups (LC, LC + DM, LC + COPD, and LC + COPD + DM; n = 10 each). Released and fluorescently labelled N-glycans were profiled by capillary electrophoresis using laser-induced fluorescence detection. Peaks with a median relative peak area of ≥1% across the complete dataset were retained for quantitative analysis, resulting in a final set of 19 peaks. Peak-wise factorial models assessed DM, COPD, and their interaction while accounting for age and sex. When the peaks were analyzed individually, Peak 12, tentatively assigned to A3G(4)3S(6)2 by GU database matching (median GU 7.23), was the only peak that remained significant after multiple-testing correction. Descriptively, its mean relative peak area (%) was approximately 21% higher in DM-positive patients, while the adjusted model confirmed DM association on the logit scale. Likewise, analysis of the 19-peak serum N-glycan profile revealed a significant association with DM, whereas no significant effect of COPD or DM × COPD interaction was detected. F(6)A2B showed an exploratory DM-associated signal but did not remain significant after conventional multiple-testing correction. Our findings suggest that diabetes may be associated with a modest but detectable shift in the serum N-glycome profile of patients with lung cancer, with A3G(4)3S(6)2 representing the strongest structure-specific finding in this cohort. However, please note that under the more stringent joint correction across all 57 peak-effect tests, the HC3-based Peak 12 result did not reach the conventional significance threshold (FDR q = 0.055).

## Simple Summary

Patients with lung cancer often have coexisting chronic diseases, including diabetes mellitus and chronic obstructive pulmonary disease. These comorbidities may influence serum N-glycan profiles and complicate the interpretation of cancer-related biomarkers. In this preliminary study, serum samples from 40 patients with lung cancer were evaluated across four balanced groups based on the presence or absence of diabetes and chronic obstructive pulmonary disease. Comorbid diabetes was associated with a modest alteration in the overall serum N-glycan profile, with a higher relative area percentage of the A3G(4)3S(6)2 glycan. In contrast, no detectable COPD-associated effect was observed in the serum N-glycan profile in this relatively limited cohort. These findings suggested that, in lung cancer patients, diabetes may represent a relevant source of variation in serum N-glycan profiles, whereas no comparable COPD-associated effect was apparent in the present dataset.

## 1. Introduction

Lung cancer remains one of the most frequently diagnosed malignancies and a leading cause of cancer-related mortality worldwide [1]. Patients with lung cancer often have chronic respiratory, cardiovascular, and metabolic disorders. Among these comorbidities, chronic obstructive pulmonary disease (COPD) and diabetes mellitus (DM) are particularly common and affect a substantial proportion of the global population [2,3,4,5]. Their coexistence with lung cancer presents a challenge for serum biomarker research, as circulating molecular profiles may reflect not only the tumor itself but also alterations associated with these comorbidities.

Protein glycosylation is a highly regulated post-translational modification that influences protein folding, stability, transport and cellular communication [6]. Changes in physiological and pathological states have been proven to affect the cellular pathways involved in glycan synthesis and processing, resulting in altered glycan structures on proteins [7,8,9,10]. Since a large proportion of serum proteins are glycosylated, the serum N-glycome can reflect biological changes occurring across multiple tissues, including metabolic, immune, inflammatory, and respiratory processes [6,10].

Accordingly, distinct serum and plasma N-glycosylation patterns have been described in LC, COPD, and DM [11,12,13,14,15,16,17,18,19,20,21,22,23,24,25,26]. Although the reported patterns vary among these conditions, several glycosylation features appear to be affected across them, including glycan branching, galactosylation, sialylation, and fucosylation [11,12,13,14,15,16,17,18,19,20,21,22,23]. Protein-specific glycosylation differences have also been reported, particularly in LC and COPD [12,13,14,15,16,17,18,19]. Together, these findings indicate a partial overlap in the glycosylation features associated with the three conditions.

Beyond disease-related processes, age and sex are well-documented sources of variation in the serum N-glycome, with differences reported in features such as galactosylation, sialylation, and branching across the adult lifespan [10,27,28,29]. These demographic factors may therefore contribute to differences observed between clinical groups, particularly when their age and sex distributions are not matched. For this reason, age and sex were considered as covariates in the statistical analyses.

However, it remains unclear how COPD and DM individually influence the serum N-glycan profile when they coexist with lung cancer, or whether their effects interact. Previous investigations have largely examined these conditions separately, while our earlier CE-LIF study directly comparing LC, COPD, and their comorbidity relied on pooled serum samples [11]. To fill this gap, the present preliminary study evaluated individual serum N-glycan profiles from 40 patients with LC across four balanced clinical groups: LC, LC + DM, LC + COPD, and LC + COPD + DM. Released serum N-glycans were fluorescently labelled and analyzed by capillary electrophoresis with laser-induced fluorescence detection (CE-LIF). The resulting profiles were evaluated using a 2 × 2 factorial design to distinguish the influence of DM, COPD, and their interaction (DM × COPD) while accounting for age and sex, with both individual glycan peaks and the complete N-glycan profile.

## 2. Materials and Methods

### 2.1. Study Design and Serum Specimens

Forty serum samples from patients with lung cancer were analyzed in four clinical groups: lung cancer without DM or COPD (LC), lung cancer with DM (LC + DM), lung cancer with COPD (LC + COPD), and lung cancer with both COPD and DM (LC + COPD + DM), 10 samples per group. Each group included five women and five men. Samples were obtained from the Department of Pulmonology, Borsod-Abaúj-Zemplén County Central Hospital and University Teaching Hospital in Miskolc, Hungary. All serum samples were collected with the appropriate ethical approval (approval No. 23580-1/2015/EKU [0180/15]) and written informed consent from the participants.

### 2.2. Chemicals and Reagents

Acetonitrile, tetrahydrofuran (THF), sodium cyanoborohydride (NaBH_3_CN; 1 M in THF), glycerol, dithiothreitol (DTT), and acetic acid were purchased from Sigma-Aldrich (St. Louis, MO, USA). Sodium dodecyl sulfate (SDS) and Nonidet P-40 (NP-40) were obtained from VWR (Radnor, PA, USA). Peptide-N-glycosidase F (PNGase F; 1.5 IUB units/μL) was produced in-house at the University of Pannonia (Veszprém, Hungary) as previously described [30]. The fluorescent labelling reagent 8-aminopyrene-1,3,6-trisulfonic acid (APTS; 120 mM) was obtained from BioScience Kft. (Budapest, Hungary). HPLC-grade water was used in all experiments.

### 2.3. Sample Preparation

The serum N-glycan sample-preparation workflow was adapted from the CE-LIF protocol as described in [20]. First, 2 μL of serum was diluted with 38 μL of HPLC-grade water, followed by the addition of 120 μL of acetonitrile. The mixture was vortexed, sonicated in an ultrasonic bath for 2 min, and centrifuged at 10,750× *g* (13,500 rpm; Microspin 12, Grant Instruments, Royston, UK) for 5 min. The supernatant was removed, and the precipitated proteins were resuspended in 40 μL of Tris-HCl buffer (10 mM, pH 8.0). The denaturation solution was prepared using 30 μL of Nonidet P-40 (0.5%), 5 μL of SDS (5%), and 5 μL of DTT (100 mM); 4 μL of this solution was added to each sample, followed by incubation at 70 °C for 15 min. Subsequently, 1 μL of PNGase F was added, and the samples were incubated at 37 °C for 2 h. The released N-glycans were fluorescently labelled with APTS using a reducing solution containing 160 μL THF, 40 μL NaBH_3_CN, 140 μL acetic acid, and 60 μL HPLC-grade water. For each sample, 1 μL of 120 mM APTS and 20 μL of reducing solution were added, and the reaction mixtures were incubated overnight at 37 °C with open vial caps [20]. After incubation, 100 μL of HPLC-grade water was added to the dry pellet and vortexed before analysis.

### 2.4. Capillary Electrophoresis

APTS-labelled serum N-glycans were separated on a PA 800 Plus capillary electrophoresis system (Beckman Coulter, Brea, CA, USA) with laser-induced fluorescence detection (excitation 488 nm; emission 520 nm) [31]. A 30 μm internal-diameter capillary with a total length of 40 cm and an effective length of 30 cm was used for all separations. Samples were introduced hydrodynamically at 1 psi for 5 s, and the separations were performed at 30 kV and 20 °C in normal polarity (anode at the injection side) using 150 mM caproic acid/253 mM Tris (pH 8.1) background electrolyte (referred to as CATRIS), as described for APTS-labelled carbohydrate and serum N-glycan analysis by Farsang et al. [32,33]. The glucose oligomer bracketing standard mixture was injected under identical conditions. Electrophoretic data were acquired using 32Karat software (Beckman Coulter).

### 2.5. Data Processing and Glycan Assignment

Electrophoretic data were evaluated using the PeakFit software (version 4.12; Systat Software, Inc., San Jose, CA, USA). Peaks with a median relative peak area of ≥1% across the complete dataset were retained for quantitative analysis, resulting in a final set of 19 peaks. The selected peaks were quantified by integration, and relative peak areas were expressed as percentages of the total integrated area of the selected peaks within each sample. Migration positions were converted to glucose-unit (GU) values using APTS-labelled glucose oligomer standards, and the corresponding glycan structures were tentatively assigned based on GU matching against our in-house glycan database. The in-house GU database was established using exoglycosidase digestion experiments for structural characterization of commercially available reference glycans. These reference-based assignments remain tentative for the serum peaks examined here.

### 2.6. Statistical Analysis

Descriptive statistics were first calculated for the four clinical groups. As the DM-positive groups were older than the DM-negative groups by an average of 8.3 years, and both age and sex are known to influence N-glycan profiles, these variables were included as covariates in all subsequent models. Given the preliminary sample size, covariate adjustment was limited a priori to age and sex to avoid model overparameterization. This focused adjustment strategy was chosen to preserve statistical stability in the four groups of 10 patients each; residual confounding by other clinical characteristics cannot be excluded.

Peak-wise differences were analyzed using a 2 × 2 factorial linear model, with DM and COPD as the two main factors and a DM × COPD interaction term. The interaction term was included to assess whether the effect of DM on the relative peak area depended on the presence of COPD, and conversely whether the effect of COPD differed according to DM status. The same model was fitted separately to each of the 19 glycan peaks. Relative peak areas (%) were converted to proportions and logit-transformed before model fitting. The single zero value in the final dataset was replaced with a lower bound of 1 × 10^−6^ before transformation. The model for each peak was specified as follows:

logit(*p*) = β_0_ + β_1_DM + β_2_COPD + β_3_ (DM × COPD) + β_4_Age + β_5_Sex + ε (1)

where *p* denotes the relative peak area expressed as a proportion, β_0_ is the model intercept, β_1_–β_5_ are the regression coefficients corresponding to the respective predictors, and ε represents the residual error. The DM and COPD coefficients represent their adjusted main effects, while the interaction coefficient indicates whether the effect of either comorbidity on relative peak area depends on the presence of the other. HC3 robust standard errors were additionally calculated as a sensitivity analysis [34]. Multiple testing was controlled using the Benjamini–Hochberg false discovery rate (FDR) [35] separately across the 19 peaks for each pre-specified effect (DM, COPD, and DM × COPD), treated as distinct hypothesis families. As a sensitivity analysis, BH correction was also applied jointly across all 57 peak-effect tests. Estimated marginal means were used for adjusted group-wise comparisons. As the peak-wise analyses use relative peak areas, their coefficients describe changes in the relative contribution of a peak rather than absolute glycan concentrations. In this exploratory clinical sensitivity analysis, the Peak 12 factorial model was refitted separately with additional adjustment for stage IV disease, small-cell histology, or smoking status.

To complement the peak-wise analysis, the complete 19-peak composition was evaluated after centred log-ratio (CLR) transformation [36,37]. For CLR transformation, the single zero value was replaced by half of the smallest positive peak area within the corresponding sample. Overall profile differences associated with DM, COPD and their interaction were assessed by PERMANOVA using Aitchison distances [36,38]. The DM × COPD interaction was first tested in the full factorial model using vegan::adonis2 with 9999 permutations and marginal tests (by = “margin”). As the interaction was not significant, DM and COPD main effects were subsequently evaluated in an additive model including age and sex, also using marginal tests. Principal component analysis (PCA) of CLR-transformed data was used for visualization, and homogeneity of multivariate dispersion was assessed before interpreting the PERMANOVA results.

All statistical analyses and visualizations were performed in R (version 4.6.1) using RStudio (version 2026.06.0 + 242; Posit Software, PBC, Boston, MA, USA). The analysis workflow used the readxl, dplyr, tidyr, purrr, ggplot2, patchwork, sandwich, lmtest, emmeans, vegan and writexl packages.

## 3. Results 

### 3.1. Serum N-Glycan Profiling and Peak Identification

**Figure 1:**
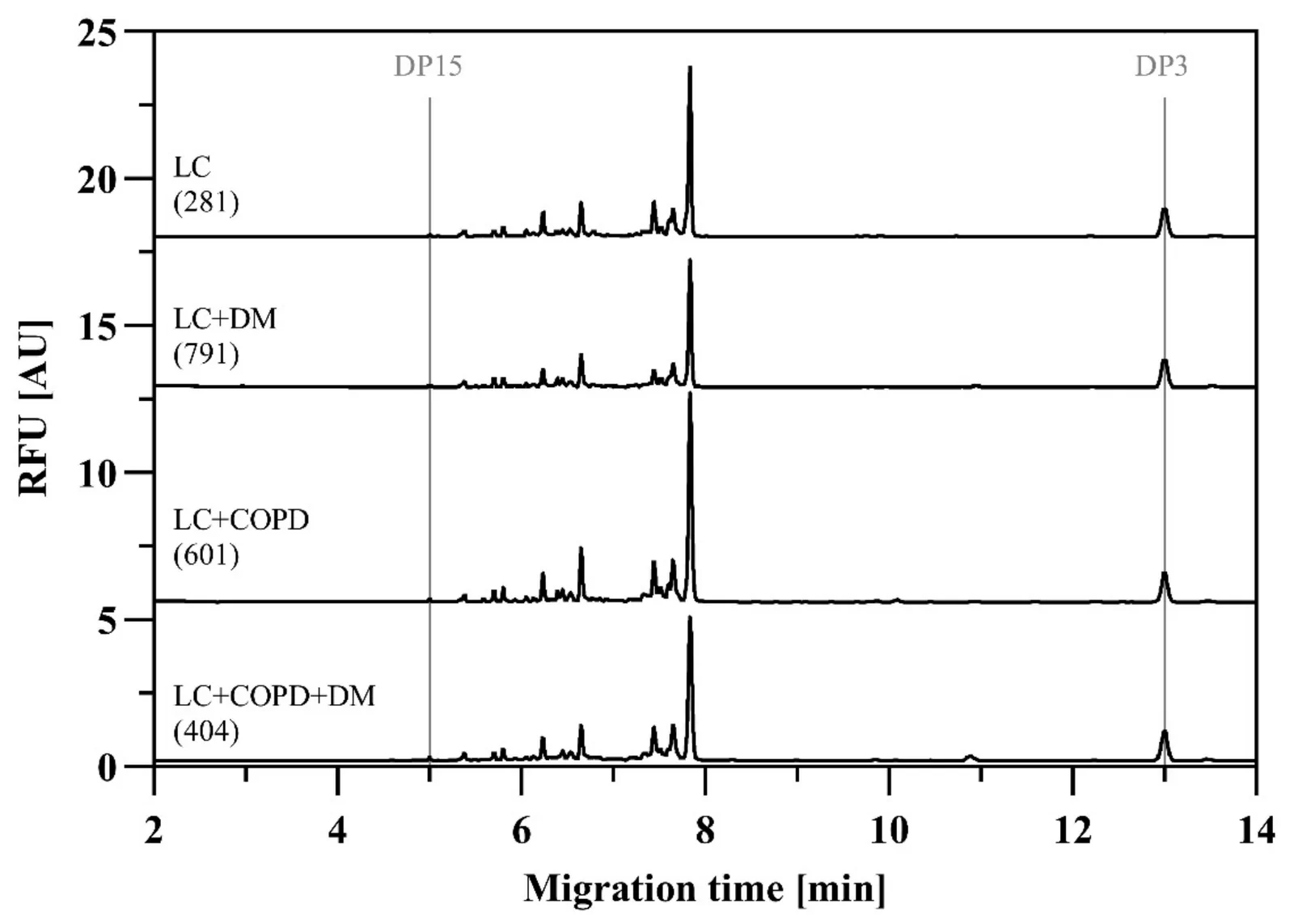
Serum N-glycan profiles obtained by CE-LIF. Each trace corresponds to an individual patient from one of the study groups, with numerical labels indicating the respective patient IDs in parentheses. DP3 and DP15 were used as reference standards for profile alignment. Separation conditions: 30 cm effective length (40 cm total length, 30 μm i.d.) capillary, 150 mM caproic acid/253 mM Tris (pH 8.1) background electrolyte (CATRIS); Applied electric potential: 30 kV (normal polarity: anode at the injection side); hydrodynamic injection at 1 psi for 5 s.

CE-LIF analysis revealed characteristic serum N-glycan profiles across the study groups. The caproic acid/Tris (CATRIS) BGE-based N-glycan separation enabled high-fidelity profiling by eliminating the need for post-labeling purification, thereby avoiding cleanup-induced glycan loss and profile bias [32]. Representative electropherograms following DP15-DP3 alignment and within-trace intensity normalization are shown in Figure 1, allowing visual comparison of the corresponding glycan profiles.

**Figure 2:**
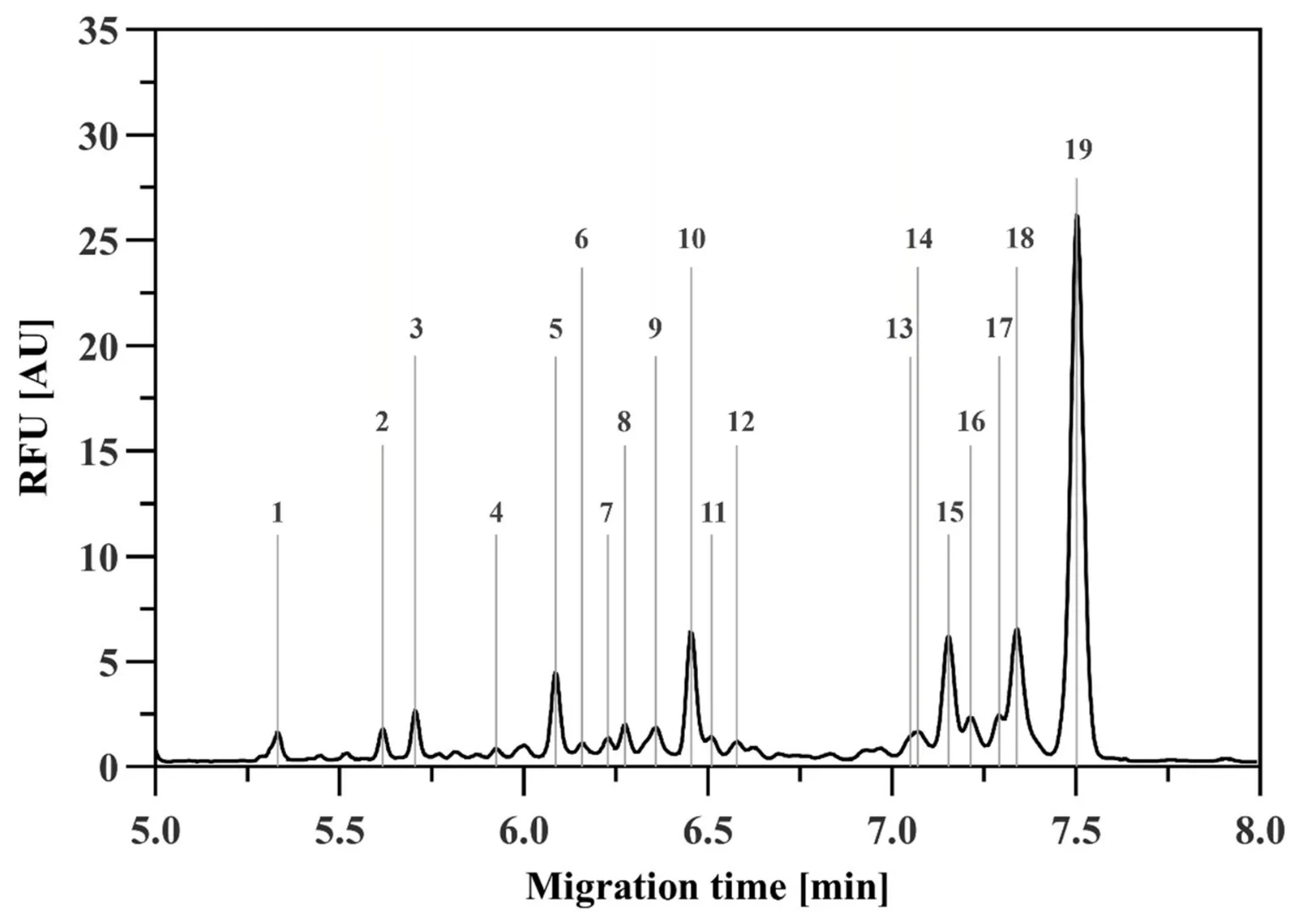
Serum N-glycan electropherogram from an LC patient (ID 281), showing the migration positions of the 19 selected peaks. Peak assignment is listed in Table 1. Conditions: same as described in Figure 1.

The electropherograms showed comparable peak patterns across the study groups, with differences mainly observed in relative signal intensities rather than in the presence or absence of individual peaks. Across the complete dataset, 19 peaks with ≥1% relative area were selected for further quantitative analysis and numbered as 1–19. Their migration positions are illustrated on the electropherogram shown in Figure 2.

The selected peaks were characterized by their median GU values and tentatively assigned to glycan structures by matching against the in-house CATRIS-GU database. Their relative peak area percentages were subsequently determined for each study group. The analytical characteristics, tentative glycan assignments, and group-wise relative peak area percentages of all 19 peaks are summarized in Table 1.

### 3.2. Cohort Characteristics and Intra-Group Profile Similarity

Demographic characteristics were compared across the study groups (Table 2). The groups were balanced by sex, whereas mean age differed significantly among them (one-way ANOVA, *p* = 0.01), with higher mean ages observed in the DM-positive groups.

Intra-group similarity of the evaluated 19-peak N-glycan profiles was assessed using complementary measures of correlation and variability. Median Spearman correlations ranged from 0.84 to 0.92, indicating generally high similarity among individual profiles. Median peak-wise coefficients of variation ranged from 25.68% to 27.66%, while median Aitchison distances ranged from 1.38 to 1.80. Together, these findings suggest that the samples within each clinical group exhibited generally consistent N-glycan profiles, providing a descriptive basis for subsequent comparisons of glycomic differences among the groups. These measures describe biological variation between patients and should not be interpreted as technical repeatability or analytical precision estimates.

### 3.3. Peak-Wise Analysis of Individual N-Glycan Peaks

**Figure 3:**
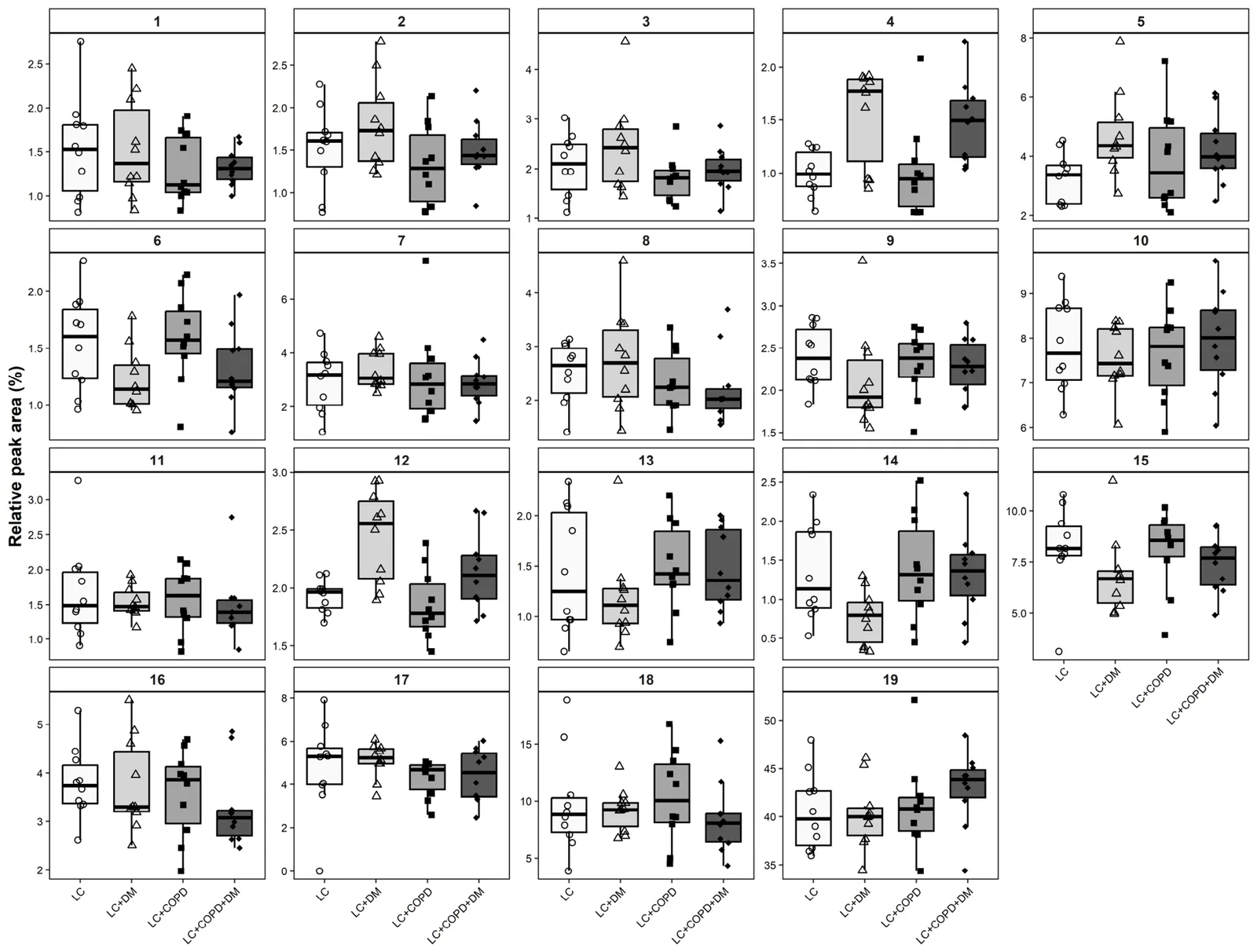
Distribution of relative peak area percentages for the 19 selected serum N-glycan peaks across the study groups. Boxes represent the median and interquartile range, with individual patient values overlaid.

Peak-wise analysis was performed to determine whether the relative area percentage of individual N-glycan peaks differed according to DM, COPD, or their interaction. The observed distributions across the study groups are shown in Figure 3, while the adjusted model results are summarized in Table 3.

The distributions of most peaks showed substantial overlap between the study groups. The clearest DM-associated difference was observed for Peak 12, while Peaks 4 and 14 also showed nominal DM-associated differences (Figure 3). These descriptive patterns were subsequently evaluated using the adjusted peak-wise models.

After adjustment for age and sex, Peak 12 was the only peak that remained significantly associated with DM after conventional multiple-testing correction (β = 0.27, FDR q = 0.01). This association was also retained using HC3 robust standard errors (HC3 FDR q = 0.02; partial η^2^ = 0.34). Peak 4 showed an HC3-based FDR-adjusted association (q = 0.047), although it was not significant under conventional FDR correction (q = 0.07); Peak 14 remained nominal only. No peak showed a significant COPD effect or DM × COPD interaction after multiple-testing correction. In the joint 57-test sensitivity analysis, Peak 12 remained significant using conventional standard errors (q = 0.037), while the HC3-based result was borderline (q = 0.055). For Peak 12, the HC3-based 95% confidence interval for the DM coefficient was 0.118–0.420. The DM coefficient remained positive in all 40 leave-one-out refits (range 0.246–0.302).

Secondary pairwise comparisons were used to examine DM- and COPD-associated differences within the corresponding clinical backgrounds. For Peak 12, significant differences were observed between LC + DM and LC (adjusted *p* < 0.01) and between LC + COPD + DM and LC + COPD (adjusted *p* = 0.03), supporting the DM-associated difference identified by the factorial model. These secondary comparisons are descriptive in relation to the primary factorial model; the nonsignificant DM × COPD interaction does not establish equivalent DM effects in the two COPD strata.

For Peak 12, the mean relative peak area was higher in the presence of DM in both COPD backgrounds. Among patients without COPD, the mean value was 2.44% in LC + DM compared with 1.93% in LC. The same direction was observed among patients with COPD, with mean values of 2.14% in LC + COPD + DM and 1.86% in LC + COPD.

Overall, Peak 12 was the only individual N-glycan peak significantly associated with DM after conventional multiple-testing correction, while no corresponding peak-specific association was identified for COPD or the DM × COPD interaction. 

### 3.4. Complete N-Glycan Profile Analysis

Analysis of the complete N-glycan profile identified a significant association with DM, which explained approximately 6% of the total compositional variation (R^2^ = 0.063, *p* = 0.019; Table 4). Sex was also associated with the profile (R^2^ = 0.060, *p* = 0.024), whereas no significant associations were observed for COPD, the DM × COPD interaction, or age. Multivariate dispersion did not differ significantly among the study groups (*p* = 0.526).

**Figure 4:**
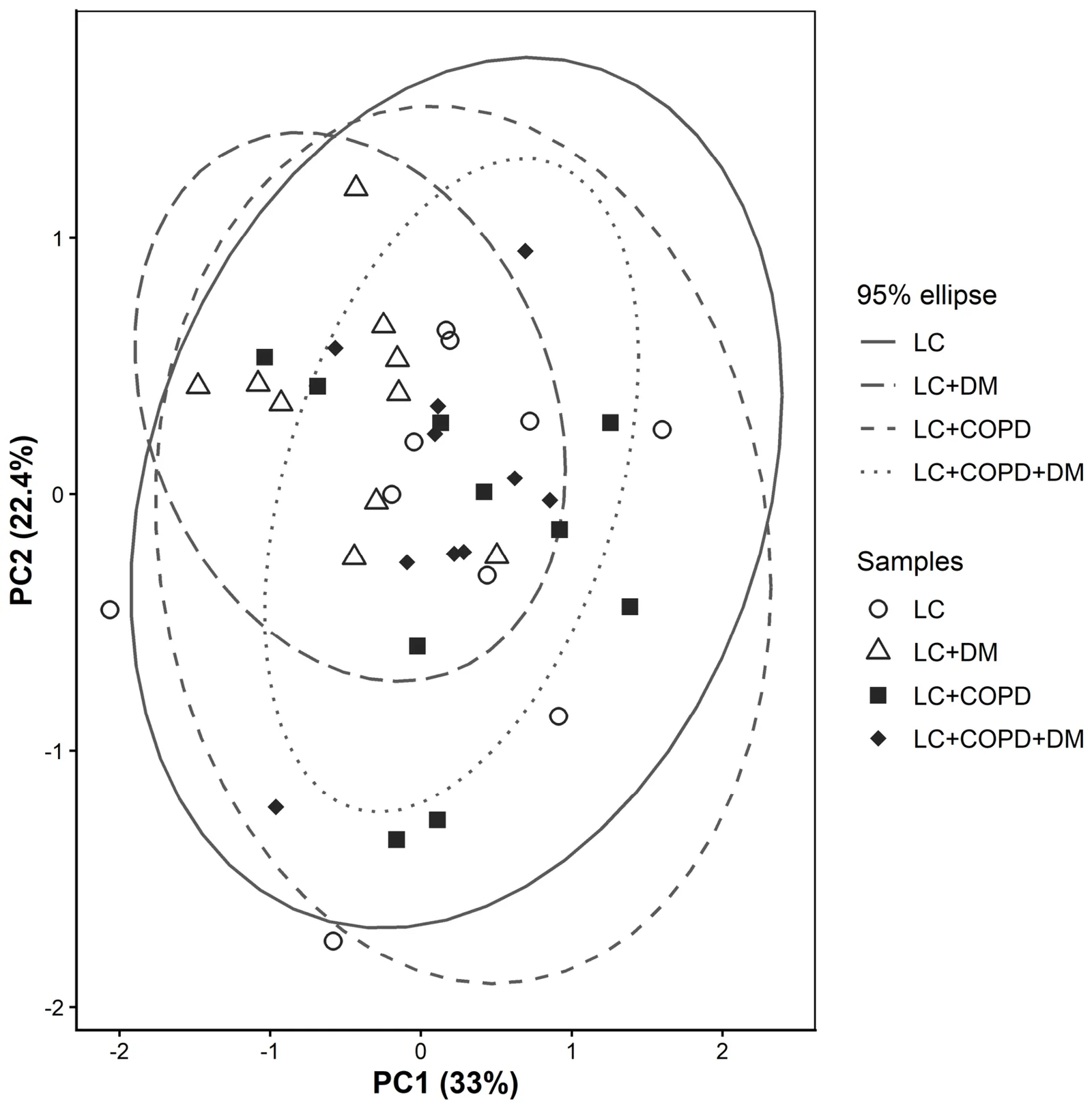
Centered CLR PCA score plot of the 19-peak serum N-glycan profiles. PC1 and PC2 accounted for 33.00% and 22.40% of total variance, respectively. Ellipses show group-level 95% regions.

The overall distribution of the N-glycan profiles across the study groups was further visualized by principal component analysis (PCA, Figure 4).

The PCA showed substantial overlap among the study groups, with no distinct group-specific clustering (Figure 4). In exploratory sensitivity models, the Peak 12 DM coefficient remained similar after separate adjustment for stage IV disease (β = 0.268), small-cell histology (β = 0.265), or smoking status (β = 0.276), compared with the primary estimate (β = 0.269). These checks do not exclude residual confounding.

## 4. Discussion

Peak 12 was tentatively assigned to the triantennary disialylated structure A3G(4)3S(6)2 by GU matching, which is consistent with previously described diabetes-associated glycosylation patterns, particularly in increased branching and sialylation [22,23]. Although COPD-associated N-glycan alterations have been reported previously [11,18,19], no corresponding COPD-associated difference was detected in the present cohort. Together with the PERMANOVA results, this indicates that the DM-associated difference involved a modest shift in the overall N-glycan profile rather than a clear division of the clinical groups. Because the cohort comprised only 40 patients, the study had limited power for smaller COPD and interaction effects, and additional clinical covariates were not incorporated to avoid overparameterization. Residual confounding by unmeasured clinical characteristics cannot be excluded, and the findings require validation in larger, clinically well-characterized cohorts.

## 5. Conclusions

In this preliminary study, lung cancer patients with coexisting DM were associated with differences in the serum N-glycan profile. Peak 12, tentatively assigned to A3G(4)3S(6)2, showed a higher relative peak area in patients with DM regardless of COPD status, and a modest DM-associated difference was also observed in the overall N-glycan profile. In contrast, no detectable independent COPD-associated difference was observed in this cohort, and there was no evidence that the DM-associated effect differed according to COPD status. These findings suggested that diabetes may contribute to serum N-glycan variation in patients with lung cancer and should be considered a potential confounding source when interpreting cancer-associated glycoprofile differences.

## Figures and Tables

**Table 1:** Analytical characteristics (median migration time and GU), glycan identification, and relative peak area percentages of the 19 selected serum N-glycan peaks across the study groups. Values for LC, LC + DM, LC + COPD, and LC + COPD + DM are presented as mean ± SD relative peak area (%).

Peak	Median Migration Time	Median GU	Glycan Identification	Relative Peak Area (%)			
				LC	LC + DM	LC + COPD	LC + COPD + DM
1	5.37	12.36	A4G(4)4S(3)1	1.53 ± 0.58	1.53 ± 0.56	1.31 ± 0.38	1.33 ± 0.21
2	5.67	10.67	F(6)A2B [6]G(4)1	1.53 ± 0.48	1.80 ± 0.53	1.32 ± 0.48	1.50 ± 0.36
3	5.75	10.24	M7D3	2.07 ± 0.62	2.45 ± 0.91	1.81 ± 0.46	1.98 ± 0.46
4	5.98	9.29	F(6)A2B	1.00 ± 0.22	1.55 ± 0.45	1.01 ± 0.44	1.48 ± 0.39
5	6.14	8.69	F(6)A2	3.25 ± 0.85	4.71 ± 1.45	3.85 ± 1.66	4.22 ± 1.18
6	6.21	8.46	F(6)A2G(4)2S(3)1	1.55 ± 0.42	1.23 ± 0.28	1.59 ± 0.40	1.32 ± 0.35
7	6.28	8.24	A2B	2.94 ± 1.13	3.38 ± 0.72	3.13 ± 1.76	2.90 ± 0.86
8	6.33	8.10	F(6)A2G(4)2S(6)1	2.51 ± 0.56	2.73 ± 0.93	2.33 ± 0.59	2.22 ± 0.69
9	6.42	7.82	A2G(4)2S(3)1	2.40 ± 0.36	2.12 ± 0.59	2.30 ± 0.39	2.28 ± 0.34
10	6.51	7.55	A2G(4)2S(6)1	7.82 ± 1.02	7.55 ± 0.75	7.66 ± 1.03	7.96 ± 1.11
11	6.57	7.41	A4G(4)4S(3)3	1.67 ± 0.68	1.53 ± 0.23	1.57 ± 0.46	1.47 ± 0.50
12	6.64	7.23	A3G(4)3S(6)2	1.93 ± 0.14	2.44 ± 0.40	1.86 ± 0.30	2.14 ± 0.34
13	7.12	6.25	Unknown	1.44 ± 0.61	1.19 ± 0.46	1.49 ± 0.44	1.47 ± 0.40
14	7.14	6.20	Unknown	1.35 ± 0.61	0.77 ± 0.35	1.39 ± 0.66	1.32 ± 0.54
15	7.22	6.07	A2[6]G(4)1S(6)1	8.21 ± 2.10	6.85 ± 1.93	8.06 ± 1.91	7.42 ± 1.42
16	7.29	5.97	A2G(4)2S(3)2	3.80 ± 0.73	3.74 ± 0.96	3.57 ± 0.90	3.28 ± 0.84
17	7.37	5.84	F(6)A2G(4)2S(6)2	4.80 ± 2.14	5.09 ± 0.82	4.28 ± 0.85	4.43 ± 1.23
18	7.41	5.78	A2(4)BG2S(6)2	9.73 ± 4.43	9.18 ± 1.88	10.32 ± 4.05	8.39 ± 3.16
19	7.58	5.55	A2(4)G2S(6)2	40.48 ± 4.03	40.16 ± 3.52	41.14 ± 4.67	42.90 ± 3.89

**Table 2:** Cohort characteristics and within-group similarity of the final 19-peak profiles. Age is presented as mean ± SD.

Group	n (F/M)	Age, Years	Median CV, %	Median Spearman ρ	Median Aitchison Distance
LC	10 (5/5)	53.20 ± 7.16	27.33	0.84	1.75
LC + DM	10 (5/5)	63.10 ± 7.91	27.66	0.92	1.61
LC + COPD	10 (5/5)	55.20 ± 7.32	25.68	0.85	1.80
LC + COPD + DM	10 (5/5)	61.90 ± 7.26	26.09	0.88	1.38

Statistical significance was defined as *p* < 0.05. The overall difference in age among the four groups was evaluated using one-way ANOVA. CV = coefficient of variation.

**Table 3:** Principal findings of the peak-wise factorial analysis.

Peak	Effect	Estimate	*p*-Value	FDR q-Value	HC3 *p*-Value	HC3 FDR q-Value
4	DM	β = 0.44	<0.01	0.07	<0.01	0.047
12	DM	β = 0.27	<0.01	0.01	<0.01	0.02
14	DM	β = −0.63	0.01	0.09	0.02	0.10

Estimates are reported on the logit scale. Nominal statistical significance was defined as *p* < 0.05, and results were considered significant after multiple-testing correction at FDR q < 0.05. Benjamini–Hochberg correction was applied across the 19 selected peaks separately for each tested effect. HC3 robust standard errors were used for sensitivity analysis.

**Table 4:** PERMANOVA results for the complete serum N-glycan profile.

PERMANOVA Term	R^2^	Permutation *p*
DM	0.063	0.019
COPD	0.031	0.240
DM × COPD interaction	0.022	0.449
Age	0.019	0.542
Sex	0.060	0.024

Statistical significance was defined as *p* < 0.05. R^2^ represents the proportion of total compositional variation explained by each variable.

## Data Availability

The data presented in this study are available upon reasonable request from the corresponding author. The data are not publicly available due to patient privacy and ethical considerations associated with the study approved by the Medical Research Council–Scientific and Research Ethics Committee of Hungary (ETT-TUKEB, Approval No. 23580-1/2015/EKU).

## Abbreviations

The following abbreviations are used in this manuscript:

ACN	acetonitrile
APTS	8-aminopyrene-1,3,6-trisulfonic acid
CE-LIF	capillary electrophoresis with laser-induced fluorescence detection
CLR	centred log-ratio
COPD	chronic obstructive pulmonary disease
COPD × DM	interaction between diabetes mellitus and chronic obstructive pulmonary disease
COPD + DM	coexistence of chronic obstructive pulmonary disease and diabetes mellitus
CV	coefficient of variation
DM	diabetes mellitus
DTT	dithiothreitol
FDR	false discovery rate
GU	glucose unit
HC3	heteroscedasticity-consistent covariance estimator, type 3
LC	lung cancer
NaBH3CN	sodium cyanoborohydride
PCA	principal component analysis
PERMANOVA	permutational multivariate analysis of variance
PNGase F	peptide-N-glycosidase F
RSD	relative standard deviation
THF	tetrahydrofuran
Tris	tris(hydroxymethyl)aminomethane

## References

[1] Sung H., Ferlay J., Siegel R.L., Laversanne M., Soerjomataram I., Jemal A., Bray F. (2021). Global Cancer Statistics 2020: GLOBOCAN estimates of incidence and mortality worldwide for 36 cancers in 185 countries. CA Cancer J. Clin..

[2] Adeloye D., Song P., Zhu Y., Campbell H., Sheikh A., Rudan I. (2022). Global, regional, and national prevalence of, and risk factors for, chronic obstructive pulmonary disease (COPD) in 2019: A systematic review and modelling analysis. Lancet Respir. Med..

[3] NCD Risk Factor Collaboration (NCD-RisC) (2024). Worldwide trends in diabetes prevalence and treatment from 1990 to 2022: A pooled analysis of 1108 population-representative studies with 141 million participants. Lancet.

[4] Islam K.M.M., Jiang X., Anggondowati T., Lin G., Ganti A.K. (2015). Comorbidity and survival in lung cancer patients. Cancer Epidemiol. Biomark. Prev..

[5] Mouronte-Roibás C., Leiro-Fernández V., Ruano-Raviña A., Ramos-Hernández C., Abal-Arca J., Parente-Lamelas I., Botana-Rial M., Priegue-Carrera A., Fernández-Villar A. (2018). Chronic obstructive pulmonary disease in lung cancer patients: Prevalence, underdiagnosis, and clinical characterization. Respiration.

[6] Reily C., Stewart T.J., Renfrow M.B., Novak J. (2019). Glycosylation in health and disease. Nat. Rev. Nephrol..

[7] Pinho S.S., Reis C.A. (2015). Glycosylation in cancer: Mechanisms and clinical implications. Nat. Rev. Cancer.

[8] Huang Y., Song J., Chen J., Li F., Zhang Y., Yang J.-H. (2025). The significance of protein N-glycosylation in the pathogenesis of lung cancer and its clinical implications. Cancer Lett..

[9] Rini J.M., Moremen K.W., Davis B.G., Esko J.D., Varki A., Cummings R.D., Esko J.D., Stanley P., Hart G.W., Aebi M., Mohnen D., Kinoshita T., Packer N.H., Prestegard J.H. (2022). Glycosyltransferases and Glycan-Processing Enzymes. Essentials of Glycobiology.

[10] Dotz V., Wuhrer M. (2019). N-glycome signatures in human plasma: Associations with physiology and major diseases. FEBS Lett..

[11] Mészáros B., Járvás G., Farkas A., Szigeti M., Kovács Z., Kun R., Szabó M., Csánky E., Guttman A. (2020). Comparative analysis of the human serum N-glycome in lung cancer, COPD and their comorbidity using capillary electrophoresis. J. Chromatogr. B.

[12] Wang Y., Liu S., Li J., Yin T., Liu Y., Wang Q., Liu X., Cheng L. (2023). Comprehensive serum N-glycan profiling identifies a biomarker panel for early diagnosis of non-small-cell lung cancer. Proteomics.

[13] Alvarez M.R.S., Zhou Q., Tena J., Lebrilla C.B., Completo G.C., Heralde F.M., Cabanatan M., Barzaga M.T., Tan-Liu N., Ladrera G.I. (2022). N-Glycan and glycopeptide serum biomarkers in Philippine lung cancer patients identified using liquid chromatography–tandem mass spectrometry. ACS Omega.

[14] Liang Y., Han P., Wang T., Ren H., Gao L., Shi P., Zhang S., Yang A., Li Z., Chen M. (2019). Stage-associated differences in the serum N- and O-glycan profiles of patients with non-small cell lung cancer. Clin. Proteom..

[15] Ruhaak L.R., Stroble C., Dai J., Barnett M., Taguchi A., Goodman G.E., Miyamoto S., Gandara D., Feng Z., Lebrilla C.B. (2016). Serum glycans as risk markers for non-small cell lung cancer. Cancer Prev. Res..

[16] Jia L., Ma T., Liang Y., Du H., Shu J., Liu X., Zhang Z., Yu H., Chen M., Li Z. (2020). Alterations in serum protein glycopatterns related to small cell lung cancer, adenocarcinoma and squamous carcinoma of the lung. RSC Adv..

[17] Balbisi M., Sugár S., Turiák L. (2026). Protein glycosylation in lung cancer from a mass spectrometry perspective. Mass Spectrom. Rev..

[18] Pavić T., Dilber D., Kifer D., Selak N., Keser T., Ljubičić Đ., Vukić Dugac A., Lauc G., Rumora L., Gornik O. (2018). N-glycosylation patterns of plasma proteins and immunoglobulin G in chronic obstructive pulmonary disease. J. Transl. Med..

[19] Komáromy A., Reider B., Járvás G., Guttman A. (2020). Glycoprotein biomarkers and analysis in chronic obstructive pulmonary disease and lung cancer with special focus on serum immunoglobulin G. Clin. Chim. Acta.

[20] Török R., Horompoly K., Szigeti M., Guttman A., Vitai M., Korányi L., Járvás G. (2021). N-Glycosylation profiling of human blood in type 2 diabetes by capillary electrophoresis: A preliminary study. Molecules.

[21] Testa R., Vanhooren V., Bonfigli A.R., Boemi M., Olivieri F., Ceriello A., Genovese S., Spazzafumo L., Borelli V., Bacalini M.G. (2015). N-Glycomic changes in serum proteins in type 2 diabetes mellitus correlate with complications and with metabolic syndrome parameters. PLoS ONE.

[22] Keser T., Gornik I., Vučković F., Selak N., Pavić T., Lukić E., Gudelj I., Gašparović H., Biočina B., Tilin T. (2017). Increased plasma N-glycome complexity is associated with higher risk of type 2 diabetes. Diabetologia.

[23] Cvetko A., Mangino M., Tijardović M., Kifer D., Falchi M., Keser T., Perola M., Spector T.D., Lauc G., Menni C. (2021). Plasma N-glycome shows continuous deterioration as the diagnosis of insulin resistance approaches. BMJ Open Diabetes Res. Care.

[24] Liu J., Dolikun M., Štambuk J., Trbojević-Akmačić I., Zhang J., Zhang J., Wang H., Meng X., Razdorov G., Menon D. (2019). Glycomics for type 2 diabetes biomarker discovery: Promise of immunoglobulin G subclass-specific fragment crystallizable N-glycosylation in the Uyghur population. OMICS.

[25] Niu J., Rodriguez E., Štambuk T., Trbojević-Akmačić I., Mraz N., Seissler J., Skurk T., Schlesinger S., Peters A., Lauc G. (2025). Longitudinal study reveals plasma glycan associations with prediabetes/type 2 diabetes in KORA study. Cardiovasc. Diabetol..

[26] Gombas V., Torok R., Vitai M., Koranyi L., Jarvas G., Guttman A., Vathy-Fogarassy A. (2025). Classifying type 2 diabetes using N-glycan profiling and machine learning algorithms. Stud. Health Technol. Inform..

[27] Knežević A., Gornik O., Polašek O., Pučić M., Redžić I., Novokmet M., Rudd P.M., Wright A.F., Campbell H., Rudan I. (2010). Effects of aging, body mass index, plasma lipid profiles, and smoking on human plasma N-glycans. Glycobiology.

[28] Knežević A., Polašek O., Gornik O., Rudan I., Campbell H., Hayward C., Wright A., Kolčić I., O’Donoghue N., Bones J. (2009). Variability, heritability and environmental determinants of human plasma N-glycome. J. Proteome Res..

[29] Krištić J., Vučković F., Menni C., Klarić L., Keser T., Beceheli I., Pučić-Baković M., Novokmet M., Mangino M., Thaqi K. (2014). Glycans are a novel biomarker of chronological and biological ages. J. Gerontol. A Biol. Sci. Med. Sci..

[30] Kovács N., Farsang R., Szigeti M., Vonderviszt F., Jankovics H. (2022). Enhanced Recombinant Protein Production of Soluble, Highly Active and Immobilizable PNGase F. Mol. Biotechnol..

[31] Farsang R., Hogyor K., Járvás G., Guttman A. (2023). Capillary zone electrophoresis of 8-aminopyrene-1,3,6-trisulfonic acid labeled carbohydrates with online electrokinetic sample cleanup. Anal. Chem..

[32] Farsang R., Járvás G., Guttman A. (2024). Purification free N-glycan analysis by capillary zone electrophoresis: Hunt for the lost glycans. J. Pharm. Biomed. Anal..

[33] Lu G., Crihfield C.L., Gattu S., Veltri L.M., Holland L.A. (2018). Capillary electrophoresis separations of glycans. Chem. Rev..

[34] Long J.S., Ervin L.H. (2000). Using heteroscedasticity consistent standard errors in the linear regression model. Am. Stat..

[35] Benjamini Y., Hochberg Y. (1995). Controlling the false discovery rate: A practical and powerful approach to multiple testing. J. R. Stat. Soc. Ser. B.

[36] Quinn T.P., Erb I., Gloor G., Notredame C., Richardson M.F., Crowley T.M. (2019). A field guide for the compositional analysis of any-omics data. GigaScience.

[37] Gloor G.B., Macklaim J.M., Pawlowsky-Glahn V., Egozcue J.J. (2017). Microbiome datasets are compositional: And this is not optional. Front. Microbiol..

[38] Anderson M.J. (2001). A new method for non-parametric multivariate analysis of variance. Austral Ecol..

